# First Trimester Tricuspid Regurgitation: Clinical Significance

**DOI:** 10.2174/1573403X19666221206115642

**Published:** 2023-03-22

**Authors:** Sofia Teixeira, Luís Guedes-Martins

**Affiliations:** 1 Instituto de Ciências Biomédicas Abel Salazar, University of Porto, Porto 4050-313 , Portugal;; 2 Centro de Medicina Fetal, Medicina Fetal Porto, Serviço de Obstetrícia-Centro Materno Infantil do Norte, Porto 4099-001, Portugal;; 3 Departamento da Mulher e da Medicina, Reprodutiva, Centro Hospitalar Universitário do Porto EPE, Centro Materno Infantil do Norte, Largo Prof. Abel Salazar, Porto 4099-001, Portugal;; 4 Unidade de Investigação e Formação-Centro Materno Infantil do Norte, Porto 4099-001, Portugal;; 5 Instituto de Investigação e Inovação em Saúde, Universidade do Porto 4200-319, Portugal

**Keywords:** Doppler, fetal echocardiography, tricuspid valve, tricuspid regurgitation, chromosomal abnormality, congenital heart defect

## Abstract

Tricuspid regurgitation is a cardiac valvular anomaly that consists of the return of blood to the right atrium during systole due to incomplete valve closure. This structure can be visualized on ultrasound between 11 and 14 weeks of gestation in most cases. Despite being a common finding, even in healthy fetuses, the presence of tricuspid regurgitation may be associated with chromosomal and structural abnormalities. The evaluation of tricuspid flow and the presence of regurgitation on first-trimester ultrasound has shown promising results regarding its role in the early detection of aneuploidies, congenital heart defects, and other adverse perinatal outcomes. This review article aims to demonstrate the importance of tricuspid regurgitation as a secondary marker, and consequently, significant benefits of its early detection when added to the combined first-trimester screening. Its value will be discussed, namely its sensitivity and specificity, alone and together with other current markers in the fetal assessment performed in the first-trimester ultrasound.

## 1 INTRODUCTION

Tricuspid regurgitation (TR) is a cardiac valvular abnormality characterized by the inability to prevent blood from flowing back to the right atrium during systole due to incomplete valve closure [1].

The tricuspid valve (TV) can be visualized between 11-14 weeks of gestation with ultrasound in 96-98% of the general population [2]. The diagnosis is based on the detection of a retrograde flow that lasts for at least half of the systole at a rate greater than 60 cm/s in a period of fetal quiescence [3]. More than one visualization attempt should be made since the regurgitant flow in the right atrium, when present, can vary its direction [4].

TR is frequently detected and is present in about 7% of healthy fetuses [5]. However, it is also associated with several cardiac and chromosomal abnormalities [6-8]. In association with the baseline parameters of the first-trimester evaluation (maternal age, nuchal translucency (NT) and maternal serum markers, free beta-hCG and PAPP-A), it detects 96% of the cases of trisomy 21 and almost all of the cases of trisomy 13, 18 and Turner syndrome [9]. There is a percentage increase in detection associated with a reduction in false positives as opposed to the general first-trimester screening [10].

Echocardiographic assessment is performed optionally after the 18^th^ week of gestation [11]. It is currently recommended in the presence of risk factors for congenital heart disease, such as a fetal NT greater than 3.5 mm [12]. Nevertheless, a large proportion of heart diseases are detected in the absence of any risk factors [12]. Thus, the value of incorporating the fetal heart is evident, which has been overlooked [10], more routinely in the analysis of these aspects to reach a diagnosis as early as possible [13, 14] with a high negative predictive [15].

TR, when present, is associated with an eight-fold increased risk of congenital heart disease [16], and when coupled with combined first-trimester screening, it can improve its performance [17].

The purpose of this review is to demonstrate the relevance of TR in the early detection of both chromosomal abnormalities and congenital heart defects. Its value has been discussed here, namely its sensitivity and specificity, used either as a standalone marker or combined with the other existing markers in the fetal evaluation performed in the first-trimester ultrasound.

## 2 METHODS

**Fig. (1):**
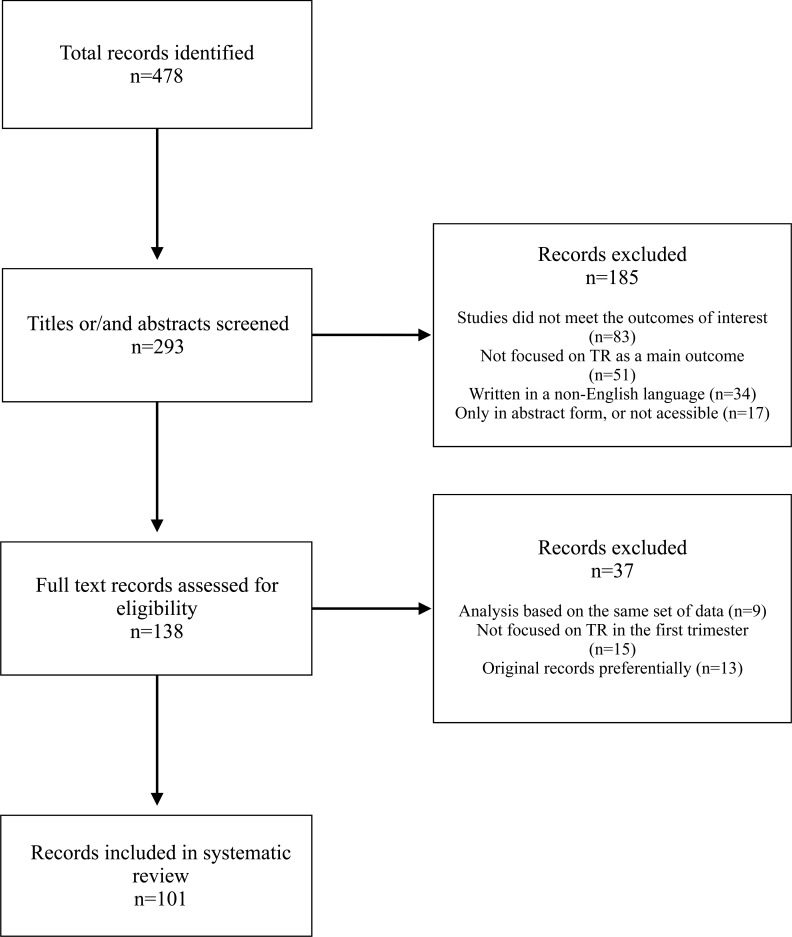
Flowchart of the search results.

To compose this review, English-language databases, predominantly PubMed, were searched using keywords, such as “doppler”, “fetal echocardiography”, “tricuspid valve”, “tricuspid regurgitation”, “chromosomal abnormality” and “congenital heart defects”, in combination primarily focused on the first trimester of pregnancy. The keywords were obtained from specific matters of the topic under study, and the references of all analyzed studies were searched to obtain the necessary information (Fig. **1**).

## 3 TRICUSPID VALVE

### 3.1 Embryology and Anatomy

Cardiac development begins towards the end of the second week post-conception [18], and the heart is not only one of the first organs to develop but one of the first to become functional [19-23].

**Fig. (2):**
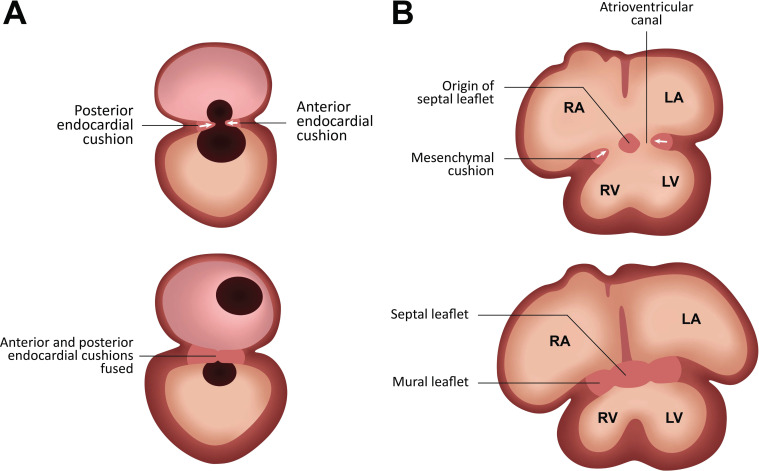
Embryology of atrioventricular valves. Valvulogenesis begins with the formation of endocardial cushions in the regions of AVC (atrioventricular canals). Two cushions form in the AVC, an anterior and a posterior, which fuse. (**A,** lateral view) A mesenchymal cushion appears on the lateral myocardial wall of the AVC, resulting in a mural leaflet. A septal leaflet emerges from the fusion of the anterior and posterior cushions. The previous structures will form the atrioventricular valves (mitral and tricuspid). (**B,** front view) RA, right atrium; LA, left atrium; RV, right ventricle; LV, left ventricle.

The first sign of valve development is the formation of endocardial cushions in the OFT and the AVC regions [24-28]. The atrioventricular valves will form by the fusion of the septal and mural leaflets [29-32]. The first ones result from the fusion of the anterior and posterior endocardial cushions in the AVC, and the second of the mesenchymal cushions arise laterally in the AVC from the myocardial ventricular wall [25, 33] (Fig. **2**).

The atrioventricular valves (mitral and tricuspid) consist of broad and asymmetric leaflets associated with a ring-shaped structure at the fixed end [27]. Despite common characteristics, each heart valve has its own distinct anatomy allowing it to function in a specific environment [34].

**Fig. (3):**
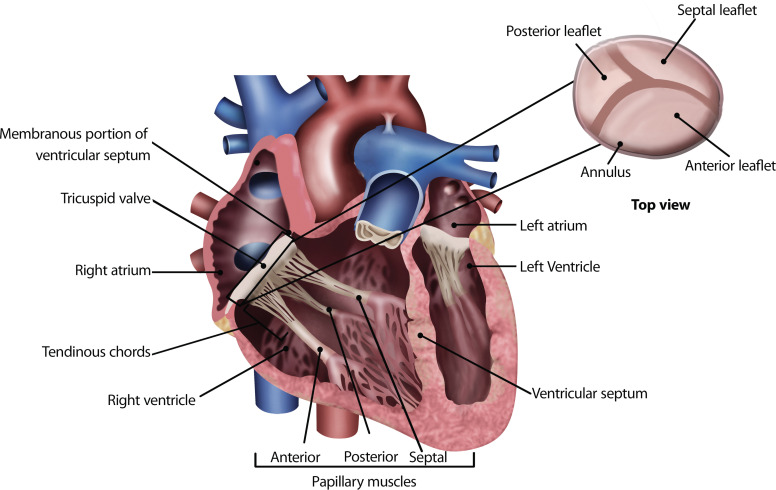
Tricuspid valve anatomy. The tricuspid valve location is between the atrium and right ventricle. It consists of three leaflets, the anterior, which extends from the infundibular area to the inferolateral wall of the right ventricle; the septal, connected to the membranous and muscular portion of the ventricular septum; and the posterior, located along the posteroinferior border of the tricuspid annulus. The valve is associated with a valvular apparatus, made up of the fibrous annulus, one of the components of the heart’s fibrous skeleton, the tendinous chords, and the papillary muscles, which are usually three: the anterior, posterior, and septal muscles.

The TV is located between the atria and the right ventricle and is associated with the valvular apparatus consisting of the fibrous annulus, the tendinous chords, and the papillary muscles [34, 35] (Fig. **3**).

The TV consists of three thin and translucent leaflets, an anterior, a posterior and a septal leaflet, all differing in size (Fig. **3**); the anterior leaflet is the largest of the three, and it goes from the infundibular area to the inferolateral wall of the right ventricle; the septal leaflet connects to the membranous and muscular portion of the ventricular septum; and the posterior leaflet, the smallest, follows the posteroinferior border of the tricuspid annulus [34]. The free margins of the leaflets connect to the tendinous chords [34]. The number of papillary muscles can vary between two to nine, but three are usually present, the anterior, posterior, and septal papillary muscles [36].

All these structures together, their specific composition and adaptability contribute to excellent and fast mobility and consequent valve function [34].

### 3.2 Protocol for Assessment of Tricuspid Flow in the First Trimester

All ultrasound assessments should rely on specific and well-defined protocols [3]. The Fetal Medicine Foundation (FMF) is recognized globally for the elaboration of standard protocols, namely those corresponding to the first-trimester evaluation [37]. Lately, the FMF has developed detailed criteria suitable for the assessment of tricuspid valve flow [37].

The learning process involves theoretical and practical exams, and in the latter, the success in some parameters is fundamental and essential, such as the identification of normal and abnormal anatomy of the fetal heart, obtaining standard images of the four cardiac chambers, the outflow tracts, the arterial duct, and cross-sectional imaging of the aortic arch [4]. It is also required to use a pulsed wave Doppler to build a color-flow mapping, which is extremely important in fetal cardiac assessment [4].

For best results, the evaluation of the flow in the tricuspid valve should be performed competently by an accredited professional in a consistent manner [38]. In about 95% of cases, it only takes five minutes for a correct evaluation of the valve flow [39].

The FMF nowadays advocates that this assessment does not need to be performed routinely on all pregnancies in the combined first-trimester screening [40]. The tricuspid flow evaluation can be reserved only for the population described as having an intermediate risk after the combined screening, *i.e*., approximately 15% of the total population [40].

In the first-trimester ultrasound, performed between 11 and 13+6 weeks of gestation, the protocol includes the visualization of the fetal heart divided into the four cardiac chambers and outflow tracts in a cross-sectional view and the assessment of flows in the tricuspid valve and the duct venosus through a color Doppler [3]. The presence or absence of TR is confirmed through a pulsed wave color Doppler [1] in the period of fetal quiescence [3].

**Fig. (4):**
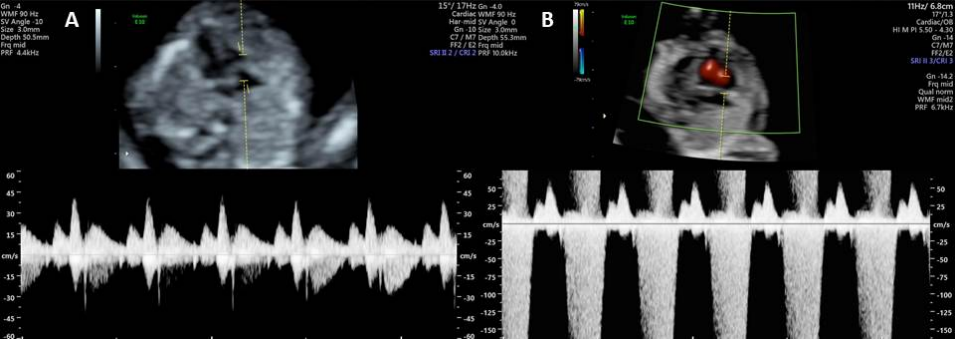
Color and pulsed Doppler examination across the tricuspid valve. Doppler velocity waveforms across the tricuspid valve at 13 weeks of gestation in a normal fetus (**A**) and in a fetus with trisomy 21 with severe tricuspid regurgitation (**B**).

A small area, sample volume, of about 2 to 3 mm is defined partially in the right atrium and across the tricuspid valve, in an apical viewing window of the four fetal heart cardiac chambers [3], with the spine in an anterior or posterior position [39]. The magnification should be sufficient for the fetal chest to occupy most of the image [41]. The positioning must be done so that the angle of the flow direction is less than 30º to the direction of the interventricular septum [40]. The sweep speed must be high (between 2 and 3 cm/s) to visualize a greater detail, which will provide a better evaluation [41] (Fig. **4**).

The tricuspid valve, as it is composed of three leaflets (any one of them or even more than one may contribute to valve insufficiency and blood flow to the right atrium), which characterizes regurgitation can be present in several directions [4] and change with each cardiac cycle [14]. Thus, the valve evaluation should be done multiple times, at least three, for complete and adequate visualization of the three valve leaflets and the jet regurgitation [3].

The presence of the defect for at least half of the systole and at a higher speed than 60 cm/s ensures the diagnosis of TR [3] (Fig. **4**). The velocity of the aortic and pulmonary blood flow at this gestational age should only reach 50 cm/s [4].

The main obstacle in Doppler evaluation is the possible overlapping of the vessels surrounding the area we intend to observe [42]. In the tricuspid valve assessment, the small area observed may include the pulmonary vessels, and a possible TR may be called into question [42]. Furthermore, pulmonary artery flow velocity reaches peaks of around 60 cm/s, and so, in these cases, if there is TR, it can only be confirmed when the valve flow velocity becomes greater and allows its distinction [42].

In addition to the pulmonary vessels, it is also important to take into account two situations in the assessment of the tricuspid valve flow: a possible overlapping of the aortic flow, which, although present as a slightly slower flow (about 30-50 cm/s) at an early gestational age, can be misinterpreted and falsely diagnosed as possible TR, and the presence of a potential slight reverse flow caused by the closure of the cusps themselves, which can also be the cause of a wrong TR diagnosis [16].

### 3.3 Fetal Tricuspid Regurgitation and First Trimester Ultrasound Screening for Fetal Aneuploidy

Most chromosomal anomalies, about 90%, are numerical and mostly autosomal trisomies (involving chromosomes 13, 16, 18, 21, 22), polyploidy, and monosomy X [43]. Trisomy 21, or Down syndrome, holds the first place in the most frequently occurring anomalies, followed by trisomy 18, or Edwards syndrome, and the third most common is trisomy 13, or Patau syndrome [44].

The risk associated with various chromosomal abnormalities increases with maternal age; however, as the probability of death in utero of aneuploid fetuses is higher than euploids, the risk decreases as the pregnancy progresses [45].

Some trisomies, such as 13 and 18, can easily be diagnosed in the prenatal period through the frequent major congenital malformations readily observable on ultrasound [43]. Trisomy 13 is associated with numerous congenital anomalies in the central nervous system (holoprosencephaly), at the craniofacial level (cleft lift and palate), and urogenital malformations (polycystic kidney) [43, 46, 47]. Trisomy 18 can affect several organs and systems, namely with growth anomalies, malformations of the skull and face (enlarged fontanels, microcephaly, triangular face shape, prominent occiput), the thorax and abdomen (short neck and sternum, umbilical or inguinal hernia), genital system (cryptorchidism, clitoral hypertrophy), extremities (clenched hands with overlapping fingers, rocker-bottom foot), central nervous system malformations (cerebellar hypoplasia, hydrocephalus, facial palsy) and cardiac anomalies, often in multiple forms (ventricular septal defects, patent ductus arteriosus) [48]. The same is not valid for trisomy 21, whose diagnosis in this period is much more difficult due to the absence of major characteristic malformations in more than 50% of cases [43].

There is significant evidence that screening for chromosomal abnormalities can be performed effectively in the first trimester [45]. Some ultrasound markers, when present, might be an indicator for chromosomal anomalies, and TR is a valuable clue [46].

Trisomy 21, or Down syndrome, is one of the most common genetic diseases, and due to its association with severe intellectual deficits and structural anomalies, the importance of its screening is valued worldwide, regardless of maternal age [49]. One of the main benefits of this screening is that it allows early detection of trisomies 18 and 13, which, paired with trisomy 21, constitute the trio of the most common chromosomal defects [50].

There are more and more options for trisomy 21 screening each day, one of which, the cell-free DNA (cffDNA) testing, has significantly improved detection efficiency; however, it is not yet available in many countries [17]. Thus, the combined first-trimester screening that results from maternal age, fetal NT, and maternal serum markers (β-hCG and PAPP-A), is still considered the best method in many countries [17]. It is simple to apply and allows for early usage [51].

This screening method makes it possible to identify a high percentage of aneuploid fetuses due to trisomy 21 and the more significant part of major chromosomal defects, namely trisomies 18 and 13, triploidy, sex chromosome aneuploidies, deletions, and unbalanced translocations [52].

The three most common trisomies are associated with increased maternal age and fetal NT, and decreased PAPP-A [50]. On the other hand, in trisomy 21, the serum-free β-hCG is elevated [53] as opposed to what happens in trisomy 18 and 13, where it decreases [50].

In situations where even serum markers are not available, particularly in developing countries, ultrasound finding can be an asset in daily clinical practice [49]. First-trimester ultrasound markers are, potentially, the most effective markers for prenatal detection of aneuploidies, reaching values above 50% [42].

The possibility of having a chromosomally abnormal fetus is common in all pregnancies and all women [45]. The individual risk of each anomaly depends on several factors, which constitute a priori risk, namely maternal age and gestational age, which, associated with the ultrasound results and maternal serological markers, determine the specific individual risk [45]. The variation from normal in the population determines the final risk estimate [42].

Screening using the combination of fetal NT and maternal serum markers allows a detection rate of trisomy 21 and other chromosomal abnormalities around 90% with a false positive rate of 5% [45]. This percentage improves if the screening includes additional ultrasound markers [17]. Among the most promising markers is the assessment of tricuspid flow and the existence of possible regurgitation using a Doppler ultrasound [17]. Its value is not only limited to the reduction of false positives from 5% to less than 3%, but it also maintains a high detection rate at around 90% [1].

The tricuspid flow was abnormal in about 56%, 33%, 30%, and 37,5% of trisomies 21, 18, 13, and Turner syndrome, respectively, and only in approximately 1% of euploid fetuses [4]. In addition, the inclusion of tricuspid flow assessment before the combined first-trimester screening allowed the detection of around 96%, 92%, 100%, and 100% of trisomies 21, 18, 13, and Turner syndrome, respectively [4]. These results are consistent with the hypothesis that TR is a potential new ultrasound marker relevant to detecting chromosomal abnormalities [54].

Most aneuploid fetuses have at least one additional abnormal marker and, due to its low prevalence in cases of euploidy, the use of additional markers, such as TR, may be a valid and alternative option to add to the classic set of markers of the combined first-trimester screening [17]. The use of second-line markers, such as TR, simultaneously with combined first-trimester screening, would thus allow an increase in accuracy and a significant impact on the diagnosis of aneuploidies if used routinely in clinical practice [50].

The high prevalence of TR in aneuploid fetuses seems to be related to the presence of high fetal NT simultaneously [4]. The existence of heart defects in fetuses with underlying chromosomal abnormalities could explain this relationship [16]. However, even in aneuploid fetuses with a heart without any pathology, a high prevalence of TR was evident in 50% of fetuses with trisomy 21 compared to about 6% in chromosomally normal fetuses [16].

The relationship between the presence of TR and the possibility of chromosomal abnormalities in the absence of a structural heart defect remains unknown [5]. However, it may be the consequence of the tricuspid valve annulus dilatation due to right ventricular dilatation [5]. It may also be related to a primary valvular defect caused by microscopic changes related to aneuploidy, such as the BMPR2 mutation usually present in fetuses with trisomy 21 and which is related to postnatal tricuspid insufficiency [5].

The benefit of using other markers, such as TR, in addition to those used in combined first-trimester screening goes beyond reducing the percentage of false positives, as it avoids unnecessary invasive diagnostic tests and, consequently, the complications inherent to them while maintaining the maximum detection level [55].

However, to include TR as a parameter of the ultrasound evaluation of chromosomal defects, it is necessary to understand the adequate training that its identification in ultrasound requires [56]. In this way, the ability to detect TR of obstetricians trained in fetal echocardiography and experienced fetal cardiologists could be evaluated [1]. In 88% and 98% of the cases, the opinion was coincident regarding the presence and absence of TR, respectively [56]. Thus, although the assessment of the fetal heart requires specific training, an obstetrician with such training may become perfectly competent to properly assess TR as a possible ultrasound marker of chromosomal abnormalities in the first trimester [1].

In the prenatal period, the definitive diagnosis of chromosomal abnormalities requires invasive methods with subsequent chromosomal analysis [55]. In addition to being quite expensive, these techniques have significant inherent risks, and therefore, with proper screening, they can and should only be performed in high-risk pregnancies [55].

### 3.4 Fetal Tricuspid Regurgitation in the First Trimester as a Marker for Congenital Heart Defects

Congenital heart defects (CHD) are the most common fetal structural defects, affecting about 8 out of every 1000 newborns [56]. Approximately one-third cause severe problems and are responsible for high morbidity and mortality in the neonatal period and childhood [57]. The assessment of fetal cardiac anatomy is usually performed in the second trimester of pregnancy, and through this screening, a prenatal detection rate of CHD of approximately 60% in affected fetuses was reported [58]. However, identifying it earlier in the first trimester may be an option to rule out cardiac anomalies without delaying the diagnosis [59].

The widespread screening of chromosomal anomalies in the first trimester contributed to an increase in the detection of CHD in that period [11]. The primary goals of the first-trimester ultrasound are confirmation of gestational age, assessment of chorionicity in multiple pregnancies, visualization of basic fetal anatomic structures [60], and screening for aneuploidies [7]. Nevertheless, the increased interest in the early detection of heart defects has become apparent [7].

Although there are several risk factors associated with CHD, such as exposure to cardioteratogenic drugs, the presence of maternal diabetes mellitus, and positive family history (previous child or parents), these standard factors only identify 10% of fetuses with heart defects [61]. In addition, the majority of cases occur in a low-risk population, and, therefore, it is necessary to develop strategies to exclude CHD early in the general population [59].

In order to achieve this purpose earlier, adequate training of professionals who perform ultrasound in this specific sense would be necessary [7]. Therefore, the first step may involve the detection of specific markers that define high-risk groups for later specialized follow-up [7].

The early identification of CHD has several benefits; it can help the family make decisions, namely about the possibility of terminating the pregnancy, earlier, safely, and more privately, and consequently, with a lower psychological impact [57]. It also allows sufficient time for a genetic evaluation, which is necessary to differentiate an isolated structural defect or, on the other hand, that contained in a genetic syndrome, along with determining the consequences that it entails [59, 61, 62].

The effectiveness of cardiac assessment in the first trimester may be affected by the small size of the fetus [14] and the fact that some anomalies are insidious and only become evident in later pregnancy [12]. Despite this, more than half of major heart defects can be detected on first-trimester ultrasound [3], and additional ultrasound markers may indirectly suggest the presence of cardiac anomalies [62]. The presence of TR is one of the examples of an easily detectable ultrasound marker that can alert to a possible CHD [7].

However, there is no solid evidence that fetal exposure in detailed ultrasound and the use of pulsed Doppler is safe for the fetus [14]. According to the International Society of Ultrasound in Obstetrics and Gynecology safety statement, the use of Doppler in the first trimester should only be reserved for the indicated cases and not the general population [63]. However, a simple cardiac screening, considering the principle, as low as reasonably achievable, allows greater effectiveness in diagnosing CHD in the first trimester and, therefore, it is a possibility to be added in clinical practice [63].

Tricuspid valve flow assessment was initially considered an additional parameter in the detection of trisomies, especially in women with an intermediate risk (between 1 in 51 and 1 in 1000) in the combined test [4]. The significant improvement in the signal quality of the high-frequency ultrasound probes allows a more detailed fetal assessment in the first trimester and, consequently, an improvement in detecting congenital anomalies in this period [64-66]. A second advantage derives from the evidence of an association between the assessment of flow across the tricuspid valve in the first trimester and the early detection of heart defects [3].

Chromosomally normal fetuses who show TR on first-trimester ultrasound are at a significantly greater risk of CHD than those who do not [41]. An 8-fold increased risk of CHD was evident in fetuses showing TR on first-trimester ultrasound [57]. In fetuses with CHD, TR was seen in about 39,2% and only 1,3% of fetuses without any anomaly [67].

Thus, TR detected in the first trimester is considered a significant risk factor for CHD in euploid fetuses, although the underlying mechanisms of this association remain unknown [41]. They may be the consequence of cardiac dysfunction, only during the first trimester, due to low compliance of the fetal heart compared to high placental resistance [41], which contributes to a reduction in diastolic function despite the high afterload at this gestational age [57]. Similarly, the presence of a normal tricuspid flow is an important protective factor [41].

The significant association between TR and the presence of CHD is evident in fetuses at high risk of CHD, namely those with an increased NT [41]. On the other hand, this association is not observed in the population at low risk for heart defects, showing a low predictive value in isolation but significant importance indirectly as a risk factor that predisposes to increased risk of CHD [41].

Standard routine ultrasound screenings have essential flaws in the detection of CHD, and for this reason, it is necessary to improve the methods for detecting high-risk cases for later referral to specialists [67]. The decision to perform echocardiography later in cases of normal karyotype, but after detection of TR, should be evaluated individually and according to the various risk factors present [41].

Thus, a critical step in detecting CHD would be the systematic investigation of TR and not just the measurement of NT in the definition of high-risk groups as the only risk criterion for referral to detailed fetal echocardiography, which is not the case in most countries yet [57]. Ideally, all fetuses with TR and/or increased NT would benefit from early fetal echocardiography, because this would result in screening 4% of the population that comprises 52% of major CHD [57]. Consequently, it would allow an earlier diagnosis; however, it would also require strict guidelines that ensure uniformity of care and the existence of qualified personnel [68].

Evaluation of TR in the first trimester is possible in approximately 98% of cases [49]. However, according to the FMF, it should only be performed in the population with an intermediate risk after combined screening [41]. Therefore, there are currently no recommendations for routine fetal cardiac assessment in the presence of TR [18]. Nonetheless, fetal echocardiography should be proposed if other CHD markers are present or major CHD is suspected [41]. Currently, the proportion of the population referred and followed by a specialist in fetal echocardiography directly influences CHD detection [67].

Early detection of heart defects is possible in the first trimester; regardless, a detailed examination requires highly qualified professionals in early fetal echocardiography [7]. The strategy will be to use markers, such as TR, to define high-risk groups that may benefit from specialized and detailed echocardiography in the first trimester, allowing for earlier suspicion and/or detection, with all the benefits that this entails [7].

### 3.5 Fetal Tricuspid Regurgitation in the First Trimester as a Predictor of Perinatal Outcomes

The importance of tricuspid valve flow assessment and the presence or absence of regurgitation has been evident in several aspects, namely in detecting cardiac anomalies and chromosomal defects in the first trimester of pregnancy [1, 67]. Although it can also be present even in the postnatal period, in newborns, children, or adults associated with other clinical conditions [68, 69] or even just as an isolated finding [70], its role is equally relevant in predicting perinatal outcomes [6].

The prevalence of TR has increased compared to the previously defined values [71]. Ultrasound methods have shown significant improvements, which allows their detection, even in less clear and significant cases, when valvular regurgitation is only located in early systole, when the jet is eccentric, or even when the regurgitant volume is significantly reduced [72].

Some studies argue that its presence is nothing more than an example of a physiological condition in early fetal development [71]. However, TR is evident when the right ventricular afterload, which represents the reflex of placental circulation and resistance, increases [73]. Fetal circulation occurs in parallel, and the function of the right ventricle is the ejection of approximately 60% of the combined ventricular output [74]. In cases of uteroplacental insufficiency, the redistribution of fetal cardiac output is evident, which privileges the left ventricle and the blood supply of the essential organs, such as the heart and brain [6]. Thus, there is an increase in the afterload of the right ventricle due to the increase in placental resistance, which may explain the presence of TR [6].

Therefore, the presence of TR in the first trimester of pregnancy may be an early marker of increased placental resistance due to placental problems, and consequently, an indicator of perinatal outcomes [6]. The existence of mild TR is negatively associated with amniotic fluid volume and fetal growth; however, there is an evident lack of research concerning this association [6].

The increase in the prevalence of TR was reported in cases of a retrograde flow in the fetal aortic isthmus due to placental insufficiency and growth restriction [73]. On the contrary, in another study, the relationship between TR and fetal growth restriction was reported as insignificant compared to those with average growth [72].

However, a recent study revealed a significant association between the presence of TR and a decrease in gestational age and fetal weight gain according to sex at birth [6]. This relationship remained present even after adjusting for some factors that could influence the result since they significantly impact the size and weight of the fetus [6]. Among these factors are maternal age, maternal weight, BMI before pregnancy and weight gain during pregnancy [75], and the presence or absence of gestational diabetes [76].

Amniotic fluid is one of many parameters used to assess fetal well-being and perinatal outcomes [77]. The factors affecting amniotic fluid volumes are complex, but its decrease is often associated with uteroplacental insufficiency and fetal oliguria [78, 79]. The ultrasound perception of reduced amniotic fluid volume has been associated with adverse perinatal outcomes [80]. In this study, the percentage of cases with values lower than the borderline amniotic fluid was higher in the group in which TR was present [6].

Other obstetric variables and perinatal outcomes have also been analyzed, namely preeclampsia and its relationship with TR, a possible consequence of placental insufficiency [6]. In these cases, trophoblastic invasion is impaired, the spiral arteries are poorly remodeled, and the capacity of the uteroplacental circulation is minimal [81]. This placental dysfunction may be the basis of preeclampsia and intrauterine growth restriction [82]. Consequently, if there is a relationship between the presence of TR and placental dysfunction, TR could be similarly related to preeclampsia, which has not been proven [6].

Thus, a relationship between the presence of TR and fetal growth and the amniotic fluid index at birth is described but unrelated to other adverse perinatal outcomes due to placental insufficiency [6]. The development of a tool that predicts future fetal growth abnormalities, amniotic fluid irregularities, or unfavorable perinatal outcomes is essential [6]. The use of TR can select cases that require a detailed investigation in late pregnancy when problems, such as fetal weight gain or amniotic fluid imbalance, may arise [6].

## 4 DISCUSSION

Ultrasound assessment is widely used in prenatal care, as it allows the assessment of fetal health even in the early stages of pregnancy [83]. In less than two decades, first-trimester ultrasound has evolved from a test limited to ensuring fetal viability, location of the pregnancy, and gestational age [84, 85] to one of the most critical tests during this period [86].

With the increase in knowledge of the natural history of congenital disabilities associated with better visualization of the fetus in early pregnancy, there is an increasing interest in the detailed ultrasound assessment of fetal anatomy simultaneously with the assessment of NT thickness in the first trimester [55].

When an early cardiac assessment is performed simultaneously with first-trimester ultrasound by experienced professionals, it only adds a few extra minutes [87], and makes it possible to detect a high number of anomalies [86].

Screening for fetal anomalies earlier and more accurately will be the goal in the future [88-90]. Bearing this objective in mind, the evaluation of secondary ultrasound markers is essential [91]. Visualization of tricuspid blood flow in the first trimester is possible in most cases [49]. Its assessment is technically challenging and requires a learning curve, so it is not routinely used in unselected populations [2].

Fetal TR is associated with numerous cardiac and extracardiac conditions [92, 93]. TR in the first trimester is closely associated with chromosomal abnormalities and represents a predictor of cardiac pathology even in the absence of karyotype defects [2]. TR is more prevalent in aneuploid and heart-defected fetuses and may be related to the prediction of pregnancy and perinatal outcomes [94-97].

In addition to its important role in the detection of anomalies, the Doppler assessment of tricuspid valve flow can reduce the number of unnecessary invasive procedures, and consequently, lead to a decrease in costs associated with a prenatal diagnosis and determination of the risk of iatrogenic missed miscarriages [95].

The experience of professionals, the selected population, the quality of the technology used, and the existence of specific guidelines for the evaluation are relevant factors to the sensitivity of this marker [98-101].

## CONCLUSION

TR is associated with several cardiac and extracardiac conditions, namely the presence of chromosomal anomalies, congenital heart defects, and other adverse perinatal outcomes.

The presence of TR in association with the markers used in combined first-trimester screening has a high positive predictive value and represents a strong marker of chromosomal abnormalities and a risk factor for heart defects. Its presence is also associated with an increased incidence of extracardiac anatomic defects and the prediction of perinatal outcomes, namely a negative influence on fetal growth and amniotic fluid volume. However, its use alone as a screening marker has low sensitivity, and thus, it represents a poor screening tool when assessed independently of the markers used in first-trimester screening.

The evaluation of the flow in the tricuspid valve and the possible detection of regurgitation is technically challenging and involves a learning curve, which is why it is not currently used in daily clinical practice. The continuous development and improvement of the imaging techniques used and the creation of specific protocols associated with adequate training of professionals are essential and allow the implementation of TR assessment in daily clinical practice. Therefore, it will be possible to detect fetal anomalies in an increasingly assertive and early way.

## LIST OF ABBREVIATIONS

TR: Tricuspid Regurgitation
TV: Tricuspid Valve
TTTS: Twin-to-Twin Transfusion Syndrome
NT: Nuchal Translucency
OFT: Outflow Tract
AVC: Atrioventricular Canal
ECM: Extracellular Matrix
EMT: Epithelial-Mesenchymal Transition
FMF: Fetal Medicine Foundation
CHD: Congenital Heart Defects
